# Exposure to air pollution increases susceptibility to ulcerative colitis through epigenetic alterations in CXCR2 and MHC class III region

**DOI:** 10.1016/j.ebiom.2024.105443

**Published:** 2024-11-13

**Authors:** Jie Chen, Han Zhang, Tian Fu, Jianhui Zhao, Jan Krzysztof Nowak, Rahul Kalla, Judith Wellens, Shuai Yuan, Alexandra Noble, Nicholas T. Ventham, Malcolm G. Dunlop, Jonas Halfvarson, Ren Mao, Evropi Theodoratou, Jack Satsangi, Xue Li

**Affiliations:** a The Second Affiliated Hospital and School of Public Health, Zhejiang University School of Medicine, Hangzhou, Zhejiang, China; b Department of Gastroenterology, The Third Xiangya Hospital, Central South University, Changsha, China; c Department of Gastroenterology, Affiliated Hangzhou First People's Hospital, Westlake University Medical College, Hangzhou, China; d Department of Pediatric Gastroenterology and Metabolic Diseases, Poznan University of Medical Sciences, 60572, Poznan, Poland; e Medical Research Council Centre for Inflammation Research, Queens Medical Research Institute, University of Edinburgh, Edinburgh, United Kingdom; f KU Leuven Department of Chronic Diseases and Metabolism, Translational Research Center for Gastrointestinal Disorders (TARGID), Leuven, Belgium; g Translational Gastro-Intestinal Unit, Nuffield Department of Medicine, John Radcliffe Hospital, Oxford, UK; h Unit of Cardiovascular and Nutritional Epidemiology, Institute of Environmental Medicine, Karolinska Institutet, Stockholm, Sweden; i Cancer Research UK Edinburgh Centre, Medical Research Council Institute of Genetics and Cancer, University of Edinburgh, Edinburgh, UK; j Department of Gastroenterology, Faculty of Medicine and Health, Örebro University, Örebro, Sweden; k Department of Gastroenterology, The First Affiliated Hospital of Sun Yat-sen University, Guangzhou, China; l Centre for Global Health Research, Usher Institute, University of Edinburgh, Edinburgh, United Kingdom

**Keywords:** Inflammatory bowel disease, Air pollution, DNA methylation, Mendelian randomization, MHC III region

## Abstract

**Background:**

This study aims to confirm the associations of air pollution with ulcerative colitis (UC) and Crohn's disease (CD); to explore interactions with genetics and lifestyle; and to characterize potential epigenetic mechanisms.

**Methods:**

We identified over 450,000 individuals from the UK Biobank and investigated the relationship between air pollution and incident inflammatory bowel disease (IBD). Cox regression was utilized to calculate hazard ratios (HRs), while also exploring potential interactions with genetics and lifestyle factors. Additionally, we conducted epigenetic Mendelian randomization (MR) analyses to examine the association between air pollution-related DNA methylation and UC. Finally, our findings were validated through genome-wide DNA methylation analysis of UC, as well as co-localization and gene expression analyses.

**Findings:**

Higher exposures to NO_x_ (HR = 1.20, 95% CI 1.05–1.38), NO_2_ (HR = 1.19, 95% CI = 1.03–1.36), PM_2.5_ (HR = 1.19, 95% CI = 1.05–1.36) and combined air pollution score (HR = 1.26, 95% CI = 1.11–1.45) were associated with incident UC but not CD. Interactions with genetic risk score and lifestyle were observed. In MR analysis, we found five and 22 methylated CpG sites related to PM_2.5_ and NO_2_ exposure to be significantly associated with UC. DNA methylation alterations at *CXCR2* and sites within the MHC class III region, were validated in genome-wide DNA methylation analysis, co-localization analysis and analysis of colonic tissue.

**Interpretation:**

We report a potential causal association between air pollution and UC, modified by lifestyle and genetic influences. Biological pathways implicated include epigenetic alterations in key genetic loci, including *CXCR2* and susceptible loci within MHC class III region.

**Funding:**

Xue Li was supported by the Natural Science Fund for Distinguished Young Scholars of Zhejiang Province (LR22H260001) and the 10.13039/501100001809National Nature Science Foundation of China (No. 82204019). ET was supported by the 10.13039/501100000289CRUK Career Development Fellowship (C31250/A22804) and the 10.13039/501100003130Research Foundation Flanders (FWO). JW was supported by Belgium by a PhD Fellowship strategic basic research (SB) grant (1S06023N). JKN was supported by the National Science Center, Poland (No. 2020/39/D/NZ5/02720). The IBD Character was supported by the European Union's Seventh Framework Programme [FP7] grant IBD Character (No. 2858546).

**Research in context**

**Evidence before this study**

Environmental factors play a role in the pathogenesis of inflammatory bowel disease (IBD), with air pollution emerging as a potential environmental risk factor. A recent analysis has revealed an elevated risk of ulcerative colitis (UC) associated with exposure to various air pollutants.

**Added value of this study**

We confirm that exposure to NO_x_, NO_2_, and PM_2.5_ are associated with increased risk of UC. Five and 22 differentially methylated sites related to PM_2.5_ and NO_2_ were significantly associated with the risk of UC. Effects of DNA methylation alterations at *CXCR2* and loci within major histocompatibility complex (MHC) class III region were further validated.

**Implications of all the available evidence**

Our study presents an exciting research framework for future investigations aiming to elucidate the potential mechanisms of environment-IBD by integrating observational, genetic, and methylation evidence. Additionally, it underscores the significance of epigenetic modifications in the complex pathogenesis of UC. The discovery of epigenetic dysregulation linking air pollution to UC risk demonstrates the translational potential of these methodologies in identifying biomarkers and characterizing pathways for pharmacological interventions.

## Introduction

Incidence rates of inflammatory bowel diseases (IBD), principally Crohn's disease (CD) and ulcerative colitis (UC), are increasing across all age groups.^1^^,^^2^ It is of particular note that the increase has been substantial in recent years^1^ in densely populated urbanized areas of newly industrialized countries. Globally, the prevalence of IBD continues to rise, and is projected to soon reach 1% in certain countries, such as the UK.^3^ Whilst genetic factors are important in IBD pathogenesis,^4^ environmental factors must underlie the recent rise in IBD incidence.^5^

We have explored a number of potential environmental risk factors in recent analyses of the UK Biobank, a cohort of approximately 500,000 adults followed prospectively since recruitment from 2006 to 2010. In particular, we have reported in detail on associations of body mass index, physical activities, sleep,^6^ diet,^7^^,^^8^ and smoking^9^ with the risk of incident IBD, and reviewed gene-environmental interactions on IBD.^10^ Air pollution merits examination as a potential factor, given its increasing impact on the environment and public health over the past decades.^11^ Epidemiological evidence has emerged to suggest a potential association between air pollution and onset of IBD.^12^, ^13^, ^14^, ^15^, ^16^ Most relevant in the present context is the recent analysis conducted within the UK Biobank and reported by Li et al. providing significant evidence of positive associations between PM_2.5_, PM_10_, NO_2_, and NO_x_ exposure and the risk of UC.^16^ However, current knowledge is incomplete, notably in the evaluation of the interactions between air pollution, genetic susceptibility and lifestyle factors in modifying the risk of IBD. Numerous studies have indicated interactions of these factors in common diseases, such as lung cancer,^17^ mortality,^18^ mental disorders,^19^, ^20^, ^21^ rheumatoid arthritis,^22^ and abdominal aortic aneurysm.^23^

Potential mechanisms involved in the effect of pollutants on disease susceptibility require exploration, and the epigenetic alterations are directly relevant. Deoxyribonucleic acid (DNA) methylation is the most widely investigated epigenetic change that could explain the impact of gene–environment interaction in disease development.^24^ Altered methylation has been suggested as a potential mediator between air pollution and a series of chronic diseases, including metabolic syndrome,^25^ cancer,^26^ and adverse respiratory health outcomes.^26^ While recent data implicate DNA methylation in the pathogenesis of IBD and the effect of smoking on IBD,^9^^,^^24^^,^^27^, ^28^, ^29^, ^30^, ^31^ its potential role in the association between air pollution and IBD has not yet been explored.

Evidence for the mechanistic importance of DNA methylation in this context has increased. A randomized controlled trial (RCT) study has found that exposure to PM_2.5_ is linked to increased levels of various inflammatory cytokines and DNA methylation in corresponding genes, and indoor air filtration can mitigate these negative effects.^32^ Mendelian randomization (MR) analysis is now accepted as a valuable complementary analytic approach for assessing the causality of associations,^33^ using genetic variants allocated randomly during human gamete formation as instrument variables, independent of potential confounders and not subject to reverse causation. Using the epigenetic MR approach, our previous study on smoking-related DNA methylation and IBD has provided an exciting research paradigm to explore potential mechanisms of environmental effects in complex diseases by integrating observational, genetic and methylation evidence.^9^

Herein, we hypothesized that a potential pathogenic effect of air pollution on IBD risk is mediated by altered DNA methylation. To test this hypothesis, we first extended the study reported by Li et al.^16^ and tested these associations. Next, we further explored possible effects of genetic susceptibility and lifestyle factors, as well as the roles of deprivation and urbanization status as interaction factors in the association between air pollution and UC development. Then, we performed MR analysis to assess potential causal association between air pollution-related DNA methylation and UC risk. Genome-wide methylation study of IBD and gene expression on colonic tissues were also conducted to finally validate the above key findings.

## Methods

### Study design

**Fig. 1:**
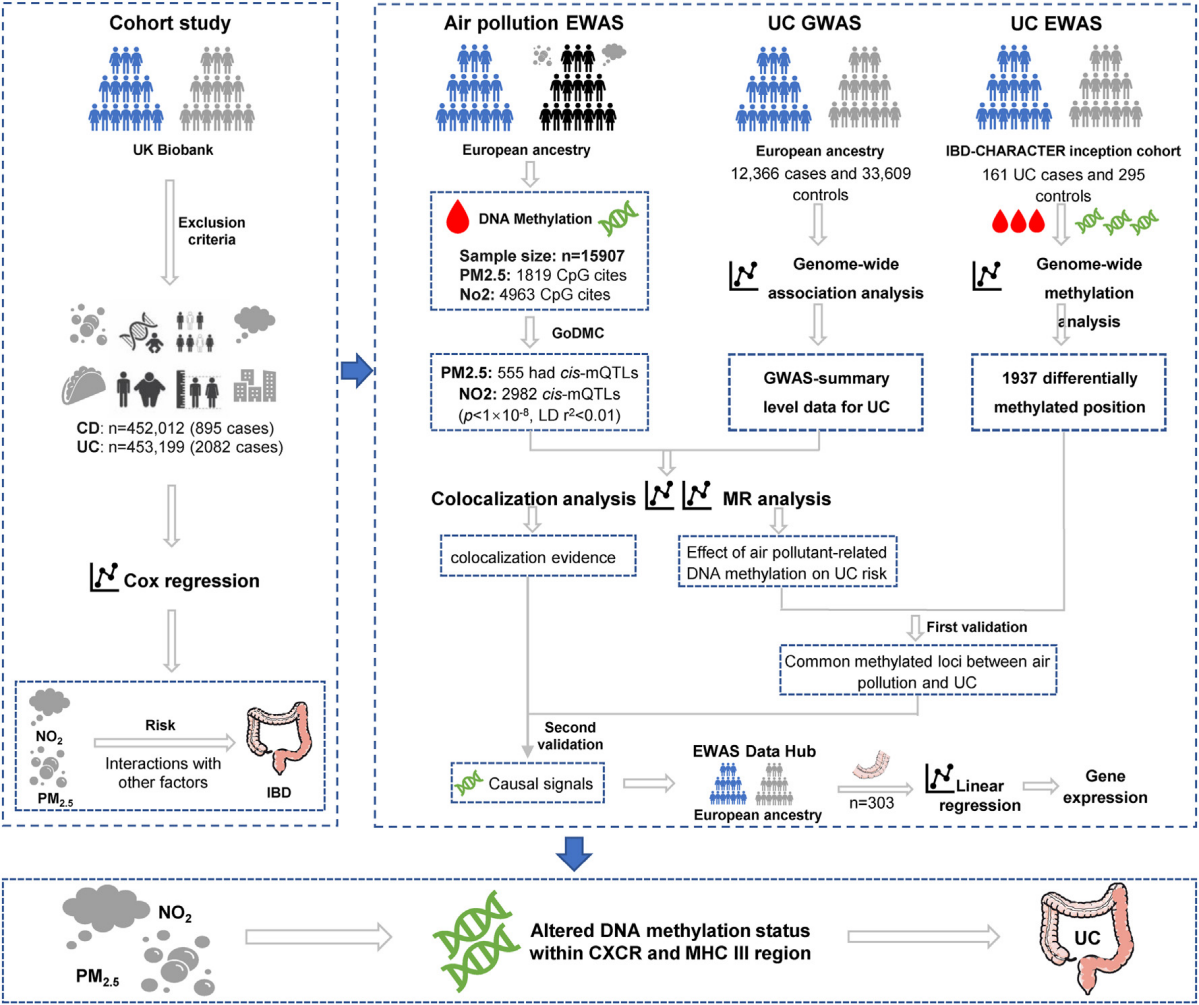
Flow chart of the study. IBD, inflammatory bowel disease; CD, Crohn's disease; UC, ulcerative colitis; PM, particulate matter; NO_x_, nitrogen oxides; NO_2_, nitrogen dioxide; EWAS, Epigenome-wide association studies; GWAS, genome-wide association study; MR, Mendelian randomization; mQTL, methylation quantitative trait loci; DNA, deoxyribonucleic acid; MHC, major histocompatibility complex; CXCR2, CXC motif chemokine receptor 2.

The study design is displayed in Fig. 1.

### Prospective cohort study

#### Study population

We used data from the UK Biobank, an ongoing national prospective cohort study with over 500,000 individuals in the UK recruited between 2006 and 2010.^34^ We excluded participants 1) without available air pollution data; 2) with pre-diagnosed IBD at baseline or incident IBD in the first year of follow-up; 3) reporting moving after recruitment. A total of 452,012 (895 cases) and 453,199 (2082 cases) participants were eligible for CD and UC analysis, respectively.

#### Assessment of air pollution

The land use regression (LUR)-based model was used to estimate the annual average concentration of particulate matter of fine inhalable particles, with diameters that are generally 2.5 μm and smaller (PM_2.5_), particulate matter of inhalable particles, with diameters that are generally 10 μm and smaller (PM_10_), nitrogen dioxide (NO_2_), and nitrogen oxides (NO_x_),^35^ as previously described (Supplementary Table S1).^35^ Previous studies compared and confirmed similar patterns of air pollution exposure in the UK Biobank cohort and the public UK Air Information Resources.^36^^,^^37^ Cumulative exposure to air pollution was calculated as pollutant concentration multiplied by the average daily time spent outdoors.^38^ The combined air pollution exposure score was estimated as follows. Firstly, a multivariate Cox regression was applied to obtain the beta value of each pollutant, then the total effect of air pollution was developed by using the formula [(β_PM2.5_ ∗ PM_2.5_ + β_PM10_ ∗ PM_10_ + β_NO2_ ∗ NO_2_ + β_NOx_ ∗ NO_x_) ∗ (4/sum of the β coefficients)].^38^ In calculation of each β value, taking β_PM2.5_ as an example, we first included participants' exposure to PM_2.5_ into the multivariable Cox proportional hazards model to estimate its effect on the risk of UC, and the partial regression coefficients for PM_2.5_ is β_PM2.5_.

#### Ascertainment of outcomes and covariates

The main endpoint of the study was the occurrence of incident IBD, including its subtypes UC and CD, throughout the follow-up period. Incident IBD were ascertained through data of primary care (converted into International Statistical Classification of Disease and Related Health Problems [ICD]-10 code), hospital inpatient (ICD-9 or ICD-10 code), and death registry (ICD-10 code). The ICD-9 and ICD-10 codes for UC were 556 and K51, while those for CD were 555 and K50. We excluded individuals with concurrent diagnoses of CD and UC when the outcomes of interest were specifically focused on either CD or UC, rather than IBD as a whole.

The following covariates collected at baseline were included in the analysis: age at recruitment, sex, ethnicity, education, household income, Townsend deprivation index (TDI), living area (urban or rural), alcohol consumption, smoking status, body mass index (BMI), physical activity, and IBD-associated healthy diet.^39^ Consuming at least 4 of the following 7 food groups (≥3 servings fruits per day; ≥3 servings vegetables per day; ≥2 servings fish per week; ≤1 serving processed meats per week; ≤1.5 servings unprocessed red meats per week; ≥3 servings whole grains per day; ≤1.5 servings refined grains per day) indicated a healthy diet. Indoor air pollution, as indicated by the presence of smokers in the household and exposure to tobacco smoke at home, as well as the use of open gas or solid fuel fires for cooking/heating, was also taken into consideration in this analysis. We assessed genetic susceptibility to UC and CD for each participant through the development of polygenic risk scores (PRS). The construction of the PRS was based on 48 and 67 independent single-nucleotide polymorphisms (SNPs) strongly associated with UC and CD (*P* < 5 × 10^−8^, r^2^ < 0.001) (the lists and specific information of the SNPs have been described in detail in our previous study),^6^ respectively, identified from a European genome-wide association study (GWAS) involving 86,640 individuals (6968 UC and 5956 CD).^40^ Then, the PRS was calculated by summing up the number of risk-increasing alleles for each SNP weighted by effect size on genetic liability, as described before.^6^ According to the PRS, the genetic risk of CD or UC in the population is divided into three levels: high (highest quintile), intermediate (quintiles 2–4) and low (the lowest quintile). Detailed information and calculation of the covariates are presented in Supplementary Table S2.

#### Statistical analysis

We first conducted correlation analyses of urbanization and TDI with air pollution. The associations between air pollution, cumulative exposure to air pollution, and combined air pollution exposure score (PM_2.5_, PM_10_, NO_2_, NO_X_, in quantiles, per interquartile range [IQR]) with the risk of IBD were estimated by performing Cox proportional hazard models. The basic model was adjusted for age, sex, and ethnicity, with the fully adjusted model further adjusted for physical activity, healthy diet, education, household income, smoking status, BMI, assessment centers, and alcohol consumption. Subsequently, the fully adjusted model was applied to evaluated the risk of UC and CD when exposed to air pollutants.

To explore the interactions between air pollution and genetics as well as lifestyle factors in the pathogenesis of UC, we conducted interaction analysis using both multiplicative and additive models.^41^ In subgroup analyses and Kaplan–Meier survival analysis, we examined differences in the risk of UC within categories of different genetic risk (low, moderate, and high), smoking status (never, previous, or current), diet quality (healthy, unhealthy), BMI (<25, ≥25 kg/m^2^), alcohol consumption (none to moderate, heavy), and physical activity (inadequate, adequate). In the joint analyses, participants were divided into several groups according to the combination of air pollution exposure score (lowest: 1st quintile; intermediate: 2nd–4th quintiles; high: 5th quintile) and genetic risk or the lifestyle factors, and taken individuals at low air pollution and low genetic risk (or never smoking; healthy diet; BMI<25 kg/m^2^, none to moderate alcohol consumption, inadequate physical activity) as the reference group to evaluated the risk of UC of other groups. We also examined whether the primary findings were changed when additionally adjusted for PRS score or indoor air pollution related variables, and whether urbanization and TDI were linked to the development of IBD.

All analyses were conducted using R software, version 4.1.3. We corrected for multiple testing using a Bonferroni corrected *P*-value threshold (0.05/5 = 0.01).

### Ethics

The UK Biobank received the ethical approval from the North West Multicenter Research Ethics Committee (REC reference: 21/NW/0157) and participants provided signed informed consent. The application number of this study is 73,595.

### Epigenetic Mendelian randomization analysis

#### Genetic instruments of air pollution-related DNA methylation

The association estimates between PM_2.5_^42^ and NO_2_^43^ and DNA methylation were derived from the corresponding genome-wide DNA methylation analyses. There are no available CpG sites or cis-mQTL for PM_10_ and NO_X_. Specifically, a total of 1819 and 4963 CpG sites were significantly differentially methylated when exposed to PM_2.5_ and NO_2_, respectively (Supplementary Tables S3–S4). Next, methylation quantitative trait loci (mQTLs), which were robustly associated with the methylation of these CpG sites, were derived from the Genetics of DNA Methylation Consortium (GoDMC).^44^ We extracted information on mQTLs including location, effect allele and other allele, and the corresponding effect estimates (including beta, se, and *P* value). Subsequently, the significant *cis*-mQTL (*P* < 1 × 10^−8^, the distance between mQTL and CpG site <1 MB) from the additive random-effects meta-analysis were extracted to proxy the methylation level for each CpG site associated with PM_2.5_ and NO_2_ for UC analysis. Finally, we performed LD pruning (r^2^ > 0.01) to filter independent instrumental variants for air pollution-related DNA methylation, and calculated the F statistic for each instrumental variable to exclude the weak instruments (F_-statistic_ < 10). The detailed information on the instrumental variables are presented in Supplementary Tables S5–S6. To elucidate potential gene pathways, methylation sites associated with air pollution were subjected to ontology analysis using the missMethyl::gomethyl function (v. 1.34.0).^45^ This allows for enrichment analysis of a gene set and indicates which molecular functions and biological processes are overrepresented. In the current ontology analysis, we input the significant CpGs, calculated the probability of genes being selected given the number of associated CpGs and tested for each gene ontology category.^46^

#### Summary-level data of UC

Genetic associations with UC were obtained from the summary-level data of a large genome-wide association study (GWAS) conducted in European ancestry.^47^ Briefly, de Lange et al. carried out a GWAS of 12,160 IBD cases and 13,145 controls and then meta-analyzed with previously published summary statistics from 12,882 IBD cases and 21,770 controls, bringing the sample size up to 45,975 participants (including 12,366 UC cases).^47^

#### MR analysis

To investigate the potential causal associations between genetically determined air pollutants-related DNA methylation and UC risk, each CpG site was seen as an exposure, and its proxy mQTLs were used as instrumental variables. The Wald ratio approach was applied to estimate the effect of DNA methylation in each CpG site on per standard deviation (SD) increment of UC risk. If there were more than one independent mQTLs for each CpG site, we pooled the estimates using the random effects inverse-variance weighted (IVW) method. We also applied the MR-Egger approach to assess potential horizontal pleiotropy.^48^ Bonferroni correction was adopted to correct for multiple testing. MR analyses were conducted in R software (version 4.1.3) by using the “TwoSampleMR” (version 0.5.6) R packages.

### Genome-wide DNA methylation and co-localization analyses

To validate the loci identified in the epigenetic MR analysis, we conducted a genome-wide DNA methylation analysis for UC, including 161 cases and 295 controls.^27^ The detailed information and procedures of genome-wide DNA methylation profiling for UC have been described elsewhere.^27^ In this analysis, we constructed a specific hypothesis based on the findings from discovery stage; as such a *P*-value threshold (<0.05) was therefore deemed acceptable and adopted for corroboration. In order to validate the proposed associations between loci exhibiting altered DNA methylation in response to air pollution and the risk of UC, we conducted a comparative analysis between the findings from epigenetic MR analysis and genome-wide methylation analysis of UC. This allowed us to identify overlapping significant gene loci that are implicated in both air pollution-induced methylation changes and UC-related methylation alterations.

We additionally conducted a co-localization analysis to explore whether the associations between the methylation level of specific CpG sites and UC risk were driven by a shared causal variant. Specifically, we extracted all available mQTLs for every significant CpG site from GoDMC and integrated them with GWAS summary-level data of UC. A summary posterior probability for the CpG site and a posterior probability for the single mQTL used as a genetic IV at 80% or higher were considered evidence of colocalization. The “coloc” R package in R software (version 4.1.3) was used to perform this analysis.^49^

### DNA methylation and gene expression, single cell RNA sequencing analysis in intestinal tissues

For those CpG sites that were validated in the genome-wide methylation analysis or were proved to have convincing evidence of colocalization, we performed linear regression to explore the regulation pattern of DNA methylation on gene expression in colonic tissue by using data provided by the EWAS Data Hub (https://ngdc.cncb.ac.cn/ewas/datahub/index). The EWAS Data Hub is a resource used to collect and normalize DNA methylation array data and archived associated data, including 75,344 samples to date, and providing reference DNA methylation profiles under different contexts, involving 81 tissues or cell types, 6 ancestry categories, and 67 diseases.^50^^,^^51^

In order to delineate the specific distribution patterns of target genes across cell subtypes, we conducted an analysis of their expression levels in RNA-seq datasets GSE150516 and GSE231993 utilizing the Seurat V5 package. These datasets comprised 8 inflamed tissues from patients with UC, 9 uninflamed tissues from self-control patients with UC, and 4 normal tissues from healthy colon samples. Cells were filtered based on the following criteria: 1) nFeature_RNA >300 and nFeature_RNA <70,000; 2) nCount_RNA <100,000; 3) percentage of mitochondria <20%; 4) percentage of red blood cells <3%. Doublet cells were identified and removed using the DoubletFinder package. Further analysis included batch effect correction using the ‘IntegrateLayers’ function with the ‘harmony’ algorithm, after normalizing and scaling UMI counts and identifying highly variable features. Integrated cells were then dimensionally reduced using the Uniform Manifold Approximation and Projection (UMAP) algorithm with a resolution of 0.5. Cell types were annotated using the SingleR package with known markers (‘ENG’, ‘PECAM1’ for endothelial cells; ‘COL14A1’, ‘COL3A1’, ‘DCN’, ‘SPARC’ for fibroblasts; ‘PHGR1’, ‘TFF3’, ‘ELF3’, ‘KRT19’, ‘CLDN3’, ‘CLDN7’ for epithelial cells; ‘CD163’, ‘CXCL8’, ‘AIF1’, ‘FCER1G’, ‘CPA3’ for myeloid cells; ‘CD79A’, ‘CD79B’, ‘MS4A1’, ‘VPREB3’ for B cells; ‘GZMA’, ‘GZMB’, ‘GZMK’, ‘PRF1’, ‘CCL5’ for NK cells; ‘CD2’, ‘IL7R’, ‘CD3E’, ‘CD3G’, ‘TRBC2’ for T cells; ‘JCHAIN’, ‘MZB1’, ‘IGHA1’, ‘DERL3’ for plasma cells). Finally, we compared the expression levels and percentages of target genes across different cell subtypes between inflamed UC tissues and normal colon tissues, and assessed significance using the Wilcoxon method.

### Role of funders

The funding sources had no role in the design of this study and did not have any role in data collection, data analyses, interpretation, writing of report, or decision to submit results.

## Results

### UK Biobank-based cohort study: air pollution was associated with incident UC but not CD

With a mean (SD) follow-up of 13.2 (2.1) years, 2082 incident UC and 895 incident CD were documented in the UK biobank cohort (Supplementary Table S7). The mean age of overall participants, individuals with UC and CD is 56.5, 57.5 and 56.9 years old. NO_X_, NO_2_, PM_10_, PM_2.5_ and the combined air pollution exposure score showed a correlation with higher urbanization (r = 0.45, 0.52, 0.37, 0.45, 0.50) and Townsend deprivation index (r = 0.44, 0.44, 0.37, 0.42, 0.47) (Supplementary Table S8). Compared with the lowest quantile of exposure, higher quantile exposure of NO_X_, NO_2_, PM_2.5_ and a combined air pollution exposure score were associated with increased risk of UC, with HR (95% CI) being 1.20 (95% CI: 1.05–1.38), 1.19 (95% CI: 1.03–1.36), 1.19 (95% CI: 1.05–1.36), 1.26 (95% CI: 1.11, 1.45) (Table 1). These associations remained consistent in all sensitivity analyses (Supplementary Table S9). In addition, the observed associations with UC remained significant when considering the cumulative exposure to air pollutants (Supplementary Table S10). No significant association between air pollutants and CD risk was observed in this prospective cohort study.

**Table 1:** Associations of air pollutants with risk of incident IBD.

	IBD				UC	*P*	CD	*P*
	Model 1	*P*	Model 2	*P*	Model 2		Model 2	
	HR (95% CI)^a^		HR (95% CI)^b^		HR (95% CI)^b^		HR (95% CI)^b^	
**NO**_**x**_								
Per IQR	1.07 (1.03, 1.11)	**1.23E-04**	1.04 (1.00, 1.08)	4.23E-02	1.06 (1.02, 1.11)	**4.04E-03**	0.97 (0.90, 1.05)	4.34E-01
Q1	Ref		Ref		Ref		Ref	
Q2	1.02 (0.91, 1.14)	7.84E-01	0.98 (0.87, 1.10)	6.88E-01	1.02 (0.89, 1.17)	7.63E-01	0.89 (0.72, 1.09)	2.53E-01
Q3	1.16 (1.04, 1.30)	**6.65E-03**	1.10 (0.98, 1.23)	9.80E-02	1.19 (1.04, 1.36)	1.13E-02	0.91 (0.74, 1.12)	3.65E-01
Q4	1.04 (0.93, 1.16)	4.83E-01	0.96 (0.86, 1.08)	5.06E-01	1.07 (0.93, 1.22)	3.53E-01	0.80 (0.64, 1.01)	5.66E-02
Q5	1.22 (1.09, 1.36)	**3.99E-04**	1.13 (1.01, 1.26)	3.87E-02	1.20 (1.05, 1.38)	**6.89E-03**	0.96 (0.78, 1.18)	6.95E-01
*P*-trend		**7.40E-04**		8.04E-02		**7.18E-03**		2.96E-01
**NO**_**2**_								
Per IQR	1.08 (1.04, 1.12)	**1.66E-04**	1.06 (1.01, 1.10)	**9.78E-03**	1.07 (1.02, 1.12)	**5.55E-03**	1.02 (0.94, 1.10)	6.78E-01
Q1	Ref		Ref		Ref		Ref	
Q2	1.09 (0.97, 1.21)	1.49E-01	1.04 (0.92, 1.16)	5.51E-01	1.06 (0.93, 1.22)	3.91E-01	0.97 (0.79, 1.21)	8.06E-01
Q3	1.14 (1.02, 1.27)	**1.97E-02**	1.08 (0.96, 1.20)	2.12E-01	1.10 (0.96, 1.25)	1.84E-01	1.03 (0.83, 1.27)	8.19E-01
Q4	1.18 (1.05, 1.31)	3.97E-03	1.09 (0.97, 1.22)	1.52E-01	1.13 (0.98, 1.29)	8.24E-02	0.99 (0.80, 1.23)	9.34E-01
Q5	1.22 (1.09, 1.36)	**6.08E-04**	1.14 (1.02, 1.28)	2.55E-02	1.19 (1.03, 1.36)	1.47E-02	1.03 (0.83, 1.29)	7.65E-01
*P*-trend		**2.14E-04**		1.81E-02		1.02E-02		7.39E-01
**PM**_**10**_								
Per IQR	1.03 (0.99, 1.07)	1.87E-01	1.01 (0.97, 1.06)	5.93E-01	1.02 (0.97, 1.08)	4.16E-01	0.99 (0.91, 1.07)	7.56E-01
Q1	Ref		Ref		Ref		Ref	
Q2	1.10 (0.98, 1.22)	9.44E-02	1.09 (0.97, 1.21)	1.47E-01	1.14 (1.00, 1.29)	5.30E-02	0.96 (0.77, 1.19)	6.89E-01
Q3	1.05 (0.94, 1.17)	3.77E-01	1.04 (0.93, 1.16)	5.41E-01	1.05 (0.92, 1.19)	5.04E-01	1.01 (0.82, 1.24)	9.37E-01
Q4	0.94 (0.84, 1.05)	2.87E-01	0.90 (0.80, 1.01)	7.40E-02	0.88 (0.76, 1.00)	5.85E-02	0.96 (0.78, 1.19)	7.07E-01
Q5	1.09 (0.97, 1.22)	1.33E-01	1.06 (0.95, 1.20)	2.86E-01	1.09 (0.95, 1.25)	2.05E-01	1.00 (0.80, 1.24)	9.75E-01
*P*-trend		9.16E-01		5.88E-01		5.33E-01		9.76E-01
**PM**_**2.5**_								
Per IQR	1.10 (1.05, 1.14)	**1.09E-05**	1.06 (1.02, 1.11)	**7.92E-03**	1.08 (1.02, 1.13)	**4.58E-03**	1.02 (0.94, 1.11)	6.44E-01
Q1	Ref		Ref		Ref		Ref	
Q2	1.02 (0.92, 1.15)	6.78E-01	0.98 (0.88, 1.10)	7.80E-01	1.02 (0.89, 1.17)	8.13E-01	0.91 (0.73, 1.12)	3.71E-01
Q3	1.07 (0.96, 1.20)	2.27E-01	1.01 (0.90, 1.13)	8.52E-01	1.06 (0.92, 1.21)	4.10E-01	0.90 (0.73, 1.12)	3.37E-01
Q4	1.18 (1.06, 1.31)	**3.06E-03**	1.11 (0.99, 1.24)	6.39E-02	1.19 (1.05, 1.36)	**8.72E-03**	0.92 (0.75, 1.14)	4.63E-01
Q5	1.20 (1.07, 1.33)	**1.31E-03**	1.10 (0.98, 1.23)	9.94E-02	1.13 (0.99, 1.29)	7.30E-02	1.03 (0.83, 1.27)	8.05E-01
*P*-trend		**6.09E-05**		1.49E-02		**7.52E-03**		7.61E-01
**Combined air pollution exposure score**								
Per IQR	1.09 (1.05, 1.14)	**1.75E-05**	1.06 (1.01, 1.10)	9.04E-03	1.08 (1.03, 1.13)	**2.58E-03**	1.00 (0.92, 1.09)	9.36E-01
Q1	Ref		Ref		Ref		Ref	
Q2	1.08 (0.97, 1.21)	1.60E-01	1.03 (0.92, 1.15)	6.51E-01	1.07 (0.93, 1.22)	3.49E-01	0.93 (0.76, 1.16)	5.33E-01
Q3	1.14 (1.02, 1.28)	1.90E-02	1.06 (0.95, 1.19)	2.88E-01	1.12 (0.98, 1.28)	9.74E-02	0.93 (0.76, 1.15)	5.25E-01
Q4	1.11 (0.99, 1.24)	7.57E-02	1.02 (0.91, 1.14)	7.49E-01	1.10 (0.96, 1.26)	1.68E-01	0.84 (0.67, 1.04)	1.08E-01
Q5	1.29 (1.16, 1.44)	**4.98E-06**	1.20 (1.07, 1.34)	**1.89E-03**	1.26 (1.11, 1.45)	**6.46E-04**	1.05 (0.85, 1.29)	6.79E-01
*P*-trend		**2.04E-05**		**6.39E-03**		**1.15E-03**		9.36E-01

IBD, inflammatory bowel disease; UC, ulcerative colitis; CD, Crohn's disease; CI, confidence interval; HR, hazard ratio; IQR, interquartile range; NO2, nitrogen dioxide; NOx, nitrogen oxides; PM, particulate matter.

*P* < 0.05 and >0.05/5 (0.01) was considered suggestive, *P* < 0.05/5 (0.01) after Bonferroni correction was considered significant and was bolded.

a Model 1 was adjusted for age, sex, ethnicity.

b Model 2 was adjusted for age, sex, ethnicity, assessment centers, household income, smoking status, education, BMI, physical activity, healthy diet, and alcohol consumption.

### Interactions between effects of air pollution, genetic susceptibility, and other lifestyle factors

In our stratification analysis, we observed that the associations between exposure to intermediate and high levels of air pollution and the risk of UC showed higher HR ratio estimates in the subgroup of participants with a high genetic risk, an unhealthy diet, a history of smoking, heavy alcohol consumption, and a higher BMI (≥25 kg/m^2^) (Table 2), in line with the association trends showed by the Kaplan–Meier plots. As shown in Supplementary Figure S1, exposure to low, intermediate and high air pollution levels showed different risk of UC in subgroup participants stratified by diet, smoking status, alcohol consumption status and physical activity (*P* log-rank <0.05), although not significant in participants with low genetic risk (*P* log-rank = 0.83), lower BMI (<25 kg/m^2^) (*P* log-rank = 0.34). Suggestive multiplicative interactions were observed for air pollution with diet (*P*_*-interaction*_ = 4.00E-02, likelihood ratio test) and alcohol consumption (*P*_*-interaction*_ = 2.79E-03, likelihood ratio test), and significant additive interactions were only observed for air pollution with genetic risk (relative excess risk due to interaction [RERI] = 0.95, 95% CI: 0.34–1.57; *P*_*-interaction*_ = 1.23E-03, likelihood ratio test) (Table 2). In joint analyses of air pollution score with genetic risk, the HR of UC showed a linear increase with the increasing levels of air pollution. Compared with individuals with low genetic risk and low air pollution level, the HRs of UC for those with high genetic risk and high air pollution level was 2.34 (95% CI: 1.70–3.23). Similarly, compared with low air pollution and healthy lifestyle (never smoking; healthy diet; BMI <25; inadequate physical activity), exposure to high levels of air pollution and unhealthy lifestyle was associated with increased risk of UC (Table 3).

**Table 2:** Interaction analyses of air pollution levels with genetic risk and lifestyle factors on the risk of incident ulcerative colitis.

	Subgroup analysis			Additive interaction	
	HR (95% CI)	*P*	*P*-interaction for multiplicative model	RERI (95% CI)	*P*-interaction for additive model
**Genetic risk**					
Low genetic risk	Ref		2.75E-01	0.95 (0.34, 1.57)	**1.23E-03**
Low air pollution level	1.02 (0.76, 1.38)	8.87E-01			
Intermediate air pollution level	0.97 (0.65, 1.44)	8.75E-01			
High air pollution level					
Intermediate genetic risk	Ref				
Low air pollution level	1.13 (0.97, 1.33)	1.22E-01			
Intermediate air pollution level	1.29 (1.06, 1.58)	1.16E-02			
High air pollution level					
High genetic risk					
Low air pollution level	Ref				
Intermediate air pollution level	1.28 (1.00, 1.63)	4.78E-02			
High air pollution level	1.64 (1.23, 2.20)	**7.92E-04**			
**Diet**					
Healthy diet			4.00E-02	0.21 (−0.12, 0.53)	1.06E-01
Low air pollution level	Ref				
Intermediate air pollution level	1.00 (0.87, 1.14)	9.81E-01			
High air pollution level	1.22 (1.04, 1.43)	1.65E-02			
Unhealthy diet					
Low air pollution level	Ref				
Intermediate air pollution level	1.37 (1.10, 1.70)	**4.47E-03**			
High air pollution level	1.42 (1.10, 1.84)	**6.48E-03**			
**BMI**					
BMI <25			1.01E-01	0.31 (0.04, 0.59)	1.25E-02
Low air pollution level	Ref				
Intermediate air pollution level	0.91 (0.75, 1.11)	3.47E-01			
High air pollution level	1.02 (0.80, 1.30)	8.68E-01			
BMI ≥25					
Low air pollution level	Ref				
Intermediate air pollution level	1.20 (1.05, 1.38)	**9.70E-03**			
High air pollution level	1.41 (1.19, 1.66)	**4.96E-05**			
**Smoking status**					
Never			2.10E-01	0.22 (−0.10, 0.55)	8.89E-02
Low air pollution level	Ref				
Intermediate air pollution level	0.99 (0.84, 1.16)	8.77E-01			
High air pollution level	1.21 (0.99, 1.48)	6.56E-02			
Ever smoked					
Low air pollution level	Ref				
Intermediate air pollution level	1.20 (1.03, 1.41)	2.17E-02			
High air pollution level	1.32 (1.10, 1.59)	**2.74E-03**			
**Alcohol consumption**					
None to moderate			**2.79E-03**	0.01 (−0.30, 0.32)	4.76E-01
Low air pollution level	Ref				
Intermediate air pollution level	1.02 (0.90, 1.16)	7.61E-01			
High air pollution level	1.27 (1.10, 1.48)	**1.64E-03**			
Heavy					
Low air pollution level	Ref				
Intermediate air pollution level	1.45 (1.12, 1.88)	**5.01E-03**			
High air pollution level	1.26 (0.92, 1.72)	1.46E-01			
**Physical activity**					
Inadequate			9.41E-01	0.05 (−0.28, 0.38)	3.83E-01
Low air pollution level	Ref				
Intermediate air pollution level	1.11 (0.91, 1.36)	3.06E-01			
High air pollution level	1.26 (0.99, 1.60)	5.60E-02			
Adequate					
Low air pollution level	Ref				
Intermediate air pollution level	1.09 (0.95, 1.25)	2.13E-01			
High air pollution level	1.27 (1.08, 1.49)	**4.76E-03**			

HR and 95% CI was calculated by Cox model adjusted for age, sex, ethnicity, assessment centers, household income, smoking status, education, BMI, physical activity, healthy diet, and alcohol consumption. Given that for all genetic-related analysis, only participants of White genetic background were included, the analysis was not adjusted for ethnicity.

BMI, body mass index; HR, hazard ratio; CI, confidence interval; RERI, relative excess risk due to interaction.

A RERI of greater than zero means positive interaction or more than additivity. A RERI of less than zero means negative interaction or less than additivity. Healthy diet indicated satisfying at least 4 of the following 7 food consumptions (≥3 servings fruits per day; ≥3 servings vegetables per day; ≥2 servings fish per week; ≤1 serving processed meats per week; ≤1.5 servings unprocessed red meats per week; ≥3 servings whole grains per day; ≤1.5 servings refined grains per day), and unhealthy diet indicated satisfying less than 4 of the following 7 food consumptions. High, intermediate, and low genetic risk indicated the highest quintile, quintiles 2–4 and lowest quintile of polygenic risk score.

*P* < 0.05 and >0.05/4 (0.0125) was considered suggestive, *P* < 0.05/4 (0.0125) after Bonferroni correction was considered significant and was bolded.

**Table 3:** Joint effects of combined air pollution levels, genetic risk and lifestyle factors for the risk of ulcerative colitis.

	Incident UC	
	HR (95% CI)	*P*
Genetic risk		
Low genetic risk		
Low air pollution level	Ref	
Intermediate air pollution level	1.03 (0.77, 1.39)	8.28E-01
High air pollution level	0.96 (0.65, 1.43)	8.50E-01
Intermediate genetic risk		
Low air pollution level	1.14 (0.85, 1.52)	3.89E-01
Intermediate air pollution level	1.30 (0.99, 1.70)	5.71E-02
High air pollution level	1.47 (1.09, 1.97)	1.04E-02
High genetic risk		
Low air pollution level	1.41 (1.01, 1.97)	4.38E-02
Intermediate air pollution level	1.77 (1.34, 2.35)	6.86E-05
High air pollution level	2.34 (1.70, 3.23)	2.27E-07
Smoking status		
Never		
Low air pollution level	Ref	
Intermediate air pollution level	0.99 (0.84, 1.16)	8.74E-01
High air pollution level	1.19 (0.98, 1.45)	8.54E-02
Ever smoked		
Low air pollution level	1.30 (1.06, 1.58)	1.05E-02
Intermediate air pollution level	1.56 (1.33, 1.83)	3.25E-08
High air pollution level	1.74 (1.45, 2.09)	2.40E-09
Diet		
Healthy diet		
Low air pollution level	Ref	
Intermediate air pollution level	1.00 (0.88, 1.14)	9.91E-01
High air pollution level	1.21 (1.03, 1.42)	1.87E-02
Unhealthy diet		
Low air pollution level	0.93 (0.74, 1.17)	5.47E-01
Intermediate air pollution level	1.27 (1.10, 1.48)	1.46E-03
High air pollution level	1.34 (1.10, 1.63)	3.63E-03
Body mass index		
BMI <25		
Low air pollution level	Ref	
Intermediate air pollution level	0.92 (0.76, 1.13)	4.30E-01
High air pollution level	1.08 (0.85, 1.36)	5.47E-01
BMI ≥25		
Low air pollution level	0.87 (0.70, 1.08)	2.14E-01
Intermediate air pollution level	1.04 (0.85, 1.26)	7.15E-01
High air pollution level	1.19 (0.96, 1.48)	1.07E-01
Alcohol consumption		
None to moderate		
Low air pollution level	Ref	
Intermediate air pollution level	1.01 (0.89, 1.15)	8.71E-01
High air pollution level	1.25 (1.08, 1.46)	3.13E-03
Heavy		
Low air pollution level	0.72 (0.56, 0.93)	1.29E-02
Intermediate air pollution level	1.09 (0.93, 1.28)	2.98E-01
High air pollution level	0.96 (0.76, 1.21)	7.38E-01
Physical activity		
Inadequate		
Low air pollution level	Ref	
Intermediate air pollution level	1.09 (0.95, 1.25)	2.01E-01
High air pollution level	1.28 (1.09, 1.50)	3.23E-03
Adequate		
Low air pollution level	1.16 (0.94, 1.44)	1.74E-01
Intermediate air pollution level	1.28 (1.10, 1.49)	1.39E-03
High air pollution level	1.44 (1.19, 1.75)	2.37E-04

BMI, body mass index; UC, ulcerative colitis; HR, hazard ratio; CI, confidence interval.

HR and 95% CI was calculated by Cox model adjusted for age, sex, ethnicity, assessment centers, household income, smoking status, education, BMI, physical activity, healthy diet, and alcohol consumption. Given that for all genetic-related analysis, only participants of White genetic background were included, the analysis was not adjusted for ethnicity.

Healthy diet indicated satisfying at least 4 of the following 7 food consumptions (≥3 servings fruits per day; ≥3 servings vegetables per day; ≥2 servings fish per week; ≤1 serving processed meats per week; ≤1.5 servings unprocessed red meats per week; ≥3 servings whole grains per day; ≤1.5 servings refined grains per day), and unhealthy diet indicated satisfying less than 4 of the following 7 food consumptions. High, intermediate, and low genetic risk indicated the highest quintile, quintiles 2–4 and lowest quintile of polygenic risk score.

*P* < 0.05 and >0.05/4 (0.0125) was considered suggestive, *P* < 0.05/4 (0.0125) after Bonferroni correction was considered significant.

In our analysis of the impact of deprivation status and urban/rural location on the risk of IBD, we observed a significant association between higher TDI and an increased risk of UC as well as CD (UC: HR_highest tertile_ = 1.21, 95% CI 1.10–1.35; CD: HR_highest tertile_ = 1.35, 95% CI 1.14–1.60). However, we did not find a significant association between living in an urban area and UC nor CD (UC: HR = 1.06, 95% CI 0.94–1.19; CD: HR = 1.08, 95% CI 0.89–1.31). The association was no longer significant when further adjusted for air pollution and smoking status (HR = 1.10, 95% CI 0.98–1.24) (Supplementary Table S11).

### Blood DNA methylation alterations of air pollutants and UC risk

**Fig. 2:**
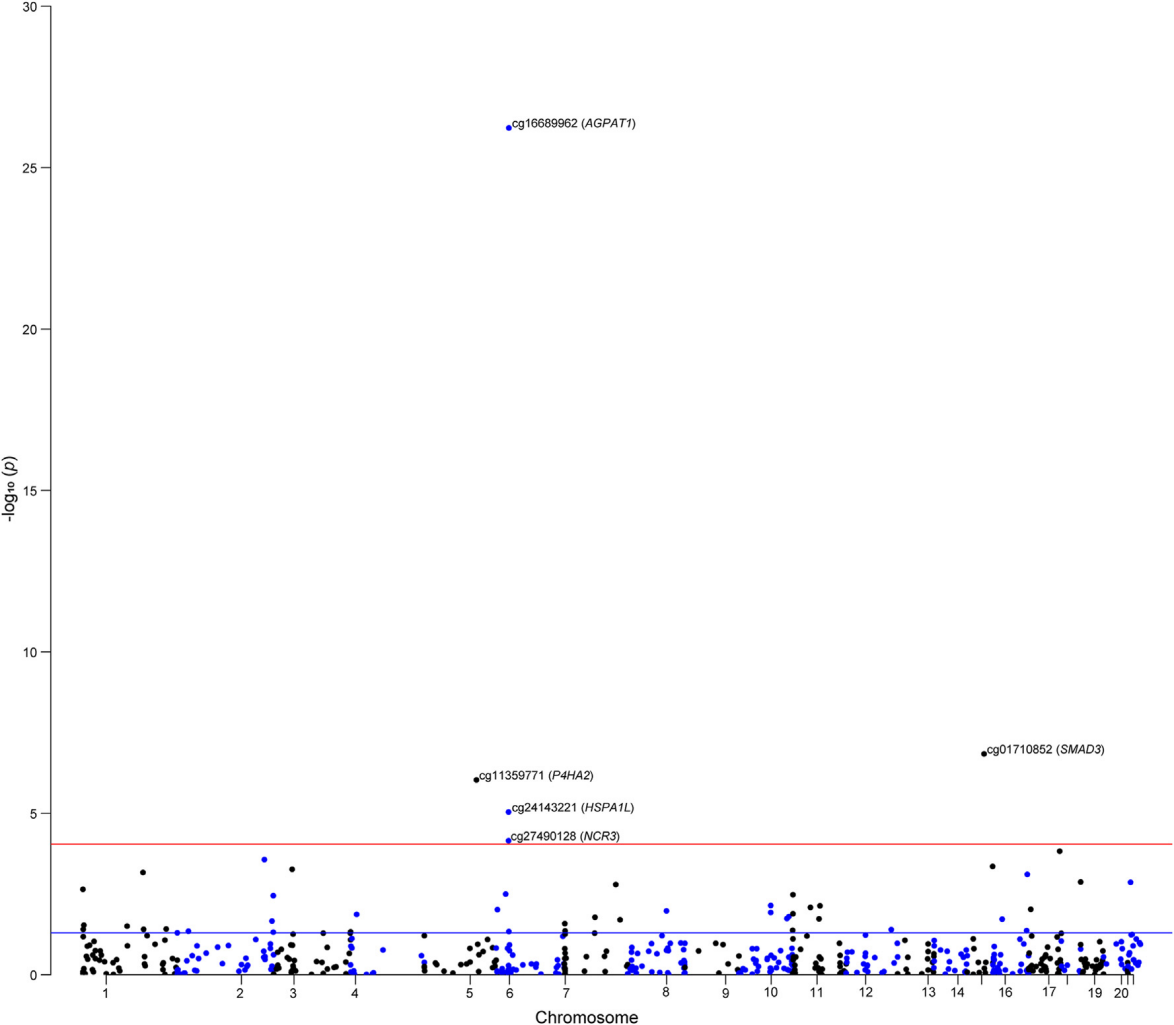
The effect of gene regulated by PM_2.5_-related DNA methylation on the risk of ulcerative colitis. The blue line means *P* = 0.05, and the red line means the threshold of FDR correction.

Subsequent analyses were exclusively focused on UC due to the significance of correlation effects observed between air pollution and UC, as opposed to CD. Among the 1819 CpG sites that were reported to be associated with PM_2.5_, 555 had available *cis*-mQTLs that could be applied as proxies in the two-sample MR analysis. We found that altered methylation at five PM_2.5_-related CpG sites was significantly associated with UC risk (*P*_*Bonferroni*_ = 0.05/555, 9.00E-05), including cg16689962 (*AGPAT1;* odds ration [OR] = 9.77, 95% CI: 6.45–14.80), cg01710852 (*SMAD3;* OR = 1.88, 95% CI: 1.49–2.38), cg11359771 (*P4HA2;* OR = 1.83, 95% CI: 1.44–2.33), cg24143221 (*HSPA1L;* OR = 0.58, 95% CI: 0.45–0.74), and cg27490128 (*NCR3;* OR = 0.41, 95% CI: 0.26–0.63) (Fig. 2, Supplementary Table S12). There was no horizontal pleiotropy for the used instruments for PM_2.5_-related DNA methylation (Supplementary Table S13).

**Fig. 3:**
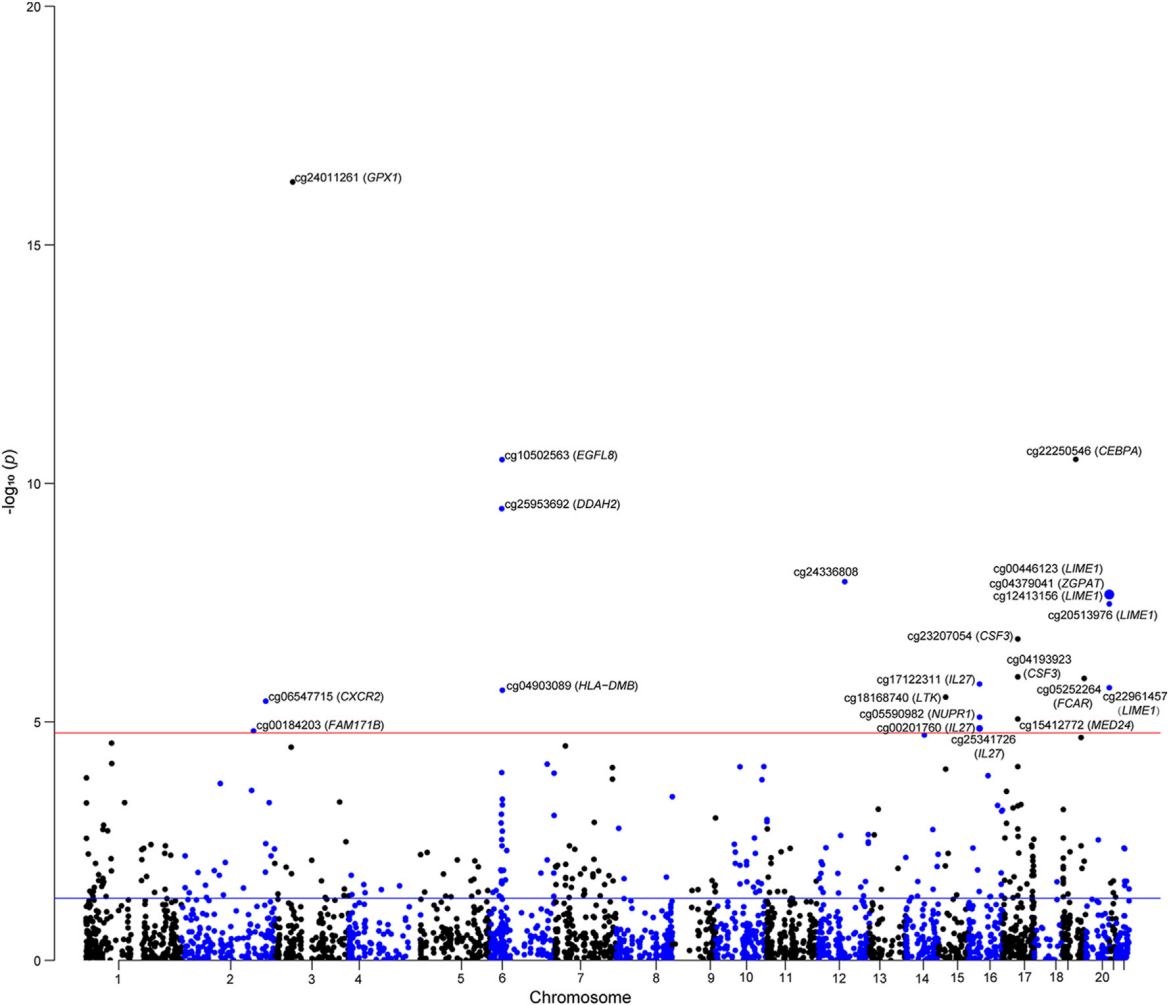
The effect of gene regulated by NO_2_-related DNA methylation on the risk of ulcerative colitis. The blue line means *P* = 0.05, and the red line means the threshold of FDR correction.

For NO_2_, 2928 of the 4963 CpG sites were discovered to have cis-mQTLs that could proxy NO_2_-related DNA methylation in the two-sample MR analysis (Supplementary Table S14). We observed that altered methylation at 22 NO_2_-related CpG sites was significantly related to UC risk (*P*_*Bonferroni*_ = 0.05/2928, 1.70E-05), including cg24011261 (*GPX1*), cg10502563 (*EGFL8*), cg22250546 (*CEBPA*), cg25953682 (*DDAH2*), cg00446123 (*LIME1*), and cg06547715 (*CXCR2*) (Fig. 3, Supplementary Table S15). Horizontal pleiotropy was observed in the instrumental variants for cg13012653 (*CAST*) and cg15818109 (*COL11A2*) (Supplementary Table S15).

In the ontology analysis of air pollution-related methylation sites, CpGs related to NO_2_ were enriched for terms pointing at the role of secretory vesicles (false discovery rate [FDR] = 2.99E-11), the lysosome (FDR = 2.30E-3), and leukocyte activation (FDR = 0.018), adaptive immunity (FDR = 2.00E-5), and T-cell receptor signaling (FDR = 4.27E-4) (Supplementary Table S16). CpG sites related to PM_2.5_ were not enriched for any biological processes.

### Validation and colocalization of differentially methylated genetic signals

**Fig. 4:**
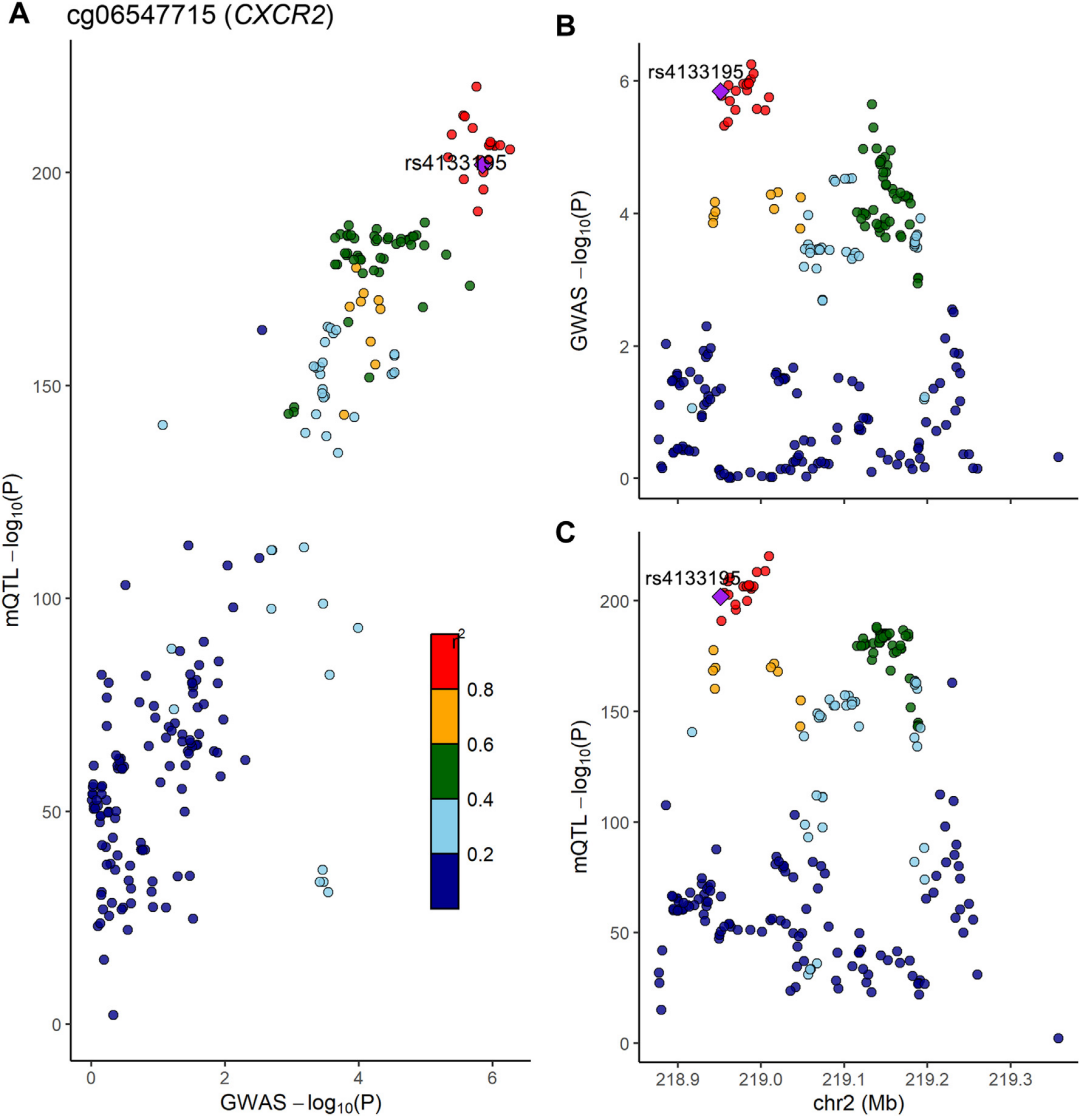
Regional plot of colocalization evidence of CpG site methylation and ulcerative colitis susceptibility. Panel A displays the *P*-value of the SNP and mQTL in corresponding GWAS and EWAS. In the Panel B and C, the horizontal axis demonstrates the base position of the SNP and mQTL, and the vertical axis is the *P*-value of the SNP and mQTL, respectively.

When comparing the epigenetic MR findings with the genome-wide DNA methylation analysis of UC (Supplementary Table S17), we successfully validated the association of DNA methylation at loci including *AGPAT1 and DDAH2* in MHC class III regions *and CXCR2* with the risk of UC (Table 4). As shown in Fig. 4 and Supplementary Table S18, we found that NO_2_-related DNA methylation at cg06547715 [*CXCR2*] had a 96.9% posterior probability of sharing a causal variant (rs4133195) with UC susceptibility. However, due to there being less than 10 mQTLs for cg16689962, we could not perform a colocalization analysis to gain evidence supporting its causal effect on UC susceptibility.

**Table 4:** The loci validated in the genome-wide methylation analysis of ulcerative colitis.

Exposure	CpG site	Gene	Beta	SE	*P*-value
PM_2.5_	cg16689962	*AGPAT1*	2.279	0.212	5.84E-27
NO_2_	cg06547715	*CXCR2*	0.096	0.021	3.68E-06

UC, ulcerative colitis; NO_2_, nitrogen dioxide; PM, particulate matter; SE, standard.

### The regulation pattern of DNA methylation on the target gene expression in colon tissue and the single cell RNA sequencing analysis

The altered DNA methylation at cg06547715 showed an effect on the expression of *CXCR2* in colonic tissues, with an effective coefficient of 1.60 (se = 0.66). In addition, the expression of genes especially in MHC class III regions was significantly regulated by the altered DNA methylation at cg16689962 (*AGPAT1:* beta = 0.65; *TNXB:* beta = 2.65; *TNF*: beta = 1.67). The scatter plots for the DNA methylation and gene expression profiles are presented in Supplementary Figure S2. To investigate the potential influence of mQTLs on the expression of their corresponding genes in intestinal tissue, we conducted a thorough inquiry using the GTEx Portal and revealed that the mQTL rs3132934 of cg16689962 serves as an eQTL for the *AGAPT1* gene in sigmoid and transverse colon tissue, with respective m-values of 0.972 and 0.981 (Supplementary Figure S3). Additionally, we performed a single-cell RNA sequencing analysis on intestinal tissue to assess the expression of these two genes across various intestinal cell types. Nine distinct cell types were identified and annotated within the intestinal tissue (Supplementary Figure S4a). The expression of *AGAPT1* and *CXCR2* was illustrated in Supplementary Figure S4b–d. It was observed that the expression of these two genes was significantly elevated in epithelial cells of patients with UC (Supplementary Figure S4c–f).

## Discussion

The current study, based on data from observational and genetic studies, defines potentially causal associations between exposure to air pollution and the development of UC and illustrates the mechanistic importance of DNA methylation in the pathophysiological pathway. Specifically, we report an observational association between air pollution and the risk of incident UC among 453,919 individuals, which are primarily driven by associations between NO_x_, NO_2_, PM_2.5_ and UC. We also report findings for the interactions between air pollution and genetic risk and specific lifestyle factors in the pathogenesis of IBD. Higher UC risk estimates were observed among groups with higher exposure to air pollution and higher genetic risk or unhealthy lifestyle (ever smoked, unhealthy diet, heavy alcohol consumption, BMI ≥25 kg/m^2^) in both subgroup and joint analyses. Subsequent MR-based analysis yielded potential causal evidence for the association between air pollution-related methylation and the risk of UC, identifying five PM_2.5_ and 22 NO_2_-related CpG sites as possible contributors. Further genome-wide methylation analysis of UC, colocalization analysis, and gene expression and single cell RNA sequencing analyses in intestinal tissues provided a hierarchy of evidence to implicate the epigenetic alterations in *CXCR2* and loci in MHC class III region on the association of air pollutants with UC. Overall, our study provides a comprehensive assessment and exploration of the association and potential mechanisms linking air pollution to UC.

Previous evidence regarding the role of air pollution in IBD pathogenesis from case–control studies conducted in the Health Improvement Network (THIN)^15^ and the European Prospective Investigation into Cancer and Nutrition cohort (EPIC)^14^ did not report any significant associations. This may be due to the case–control design and relatively small number of IBD cases (367 CD and 591 UC). In contrast, a Canada population-based cohort study revealed an association between O_x_ and increased risk of pediatric-onset IBD,^12^ and a modelling study found that PM_2.5_ environmental exposure was associated with pediatric IBD.^52^ The most recent study involving 450,000 participants, with more individuals with UC than CD, conducted in UK Biobank proposed associations between exposure to PM_2.5_, PM_10_, NO_2_, and NO_x_ and the risk of UC.^16^ Our research was prompted by and built upon this index study; we expanded upon the findings with updated data and further assessed the interactions between genetic factors, lifestyle variables, and air pollution through subgroup, interaction, and joint analyses. Additionally, we examined the impact of urbanization and deprivation. We confirmed that high levels of air pollutants are associated with increased risk of UC, and the new findings of interactions between air pollution and genetics as well as lifestyle in the development of UC. Significant interactions were observed for genetic risk, alcohol consumption, and suggestive interactions were discovered for diet and BMI in the associations of air pollutants with UC risk. This indicates that genetic risk and alcohol consumption play a more important role in the observed effects compared to other lifestyle factors. High genetic risk, an unhealthy diet, higher BMI and a personal history of smoking were implicated as effect modifiers of the association between air pollution and incident UC. Of note, participants with a high genetic risk and high air pollution exposure showed higher risk estimates than those with lowest genetic risk but high air pollution, implicating the importance of genetics in shaping the risk of UC when exposed to air pollution. Thus, the interaction and joint analyses explored how genetic and lifestyle play roles on air pollution and UC risk from different dimensions and presented preliminary evidence.

Furthermore, we demonstrate significant correlations between air pollution and urbanization, as well as between pollution and deprivation assessed by the TDI. This is consistent with the previously conducted study, which reported strong positive associations for air pollutants with urbanization and socioeconomic deprivation.^53^ The observation that the positive correlations between deprivation and the risk of UC diminish in statistical significance upon further adjustment for both air pollution and smoking status is noteworthy, suggesting that these two factors may serve as pivotal determinants within deprived areas. These findings reveal the complex associations between deprivation, air pollution and IBD. Our earlier studies of children in Scotland have highlighted this complexity as well, demonstrating CD but not UC to be associated with affluence, rather than deprivation.^54^ Further research is warranted to explore innovative potential avenues for investigating the relationship between the environment and the development of IBD. While our observations have been conducted in the UK, these findings may have direct implications for the rapid surge in IBD cases in newly industrialized nations.

The absence of any significant associations between air pollutants and the risk of developing CD is particularly noteworthy. Combining our findings with previous studies, we believe that there are several possible explanations. These include both statistical explanations based on power as well as biological explanations. Data suggest CD & UC are related to polygenic diseases–these share some genetic determinants, with others specific for each disease. We suggest a similar pattern is emerging for the exposome. The degree to which environmental risk factors are shared between CD and UC has been explored in epidemiological studies and meta-analyses.^7^^,^^55^ In our recent studies, we explored effects of a series of lifestyle factors on the development of CD and UC, respectively, and have established the positive associations of current smoking and previous smoking,^9^ unfavourable lifestyles (ever smoking, obesity, sleep <7 or >8 h/day, unhealthy dietary habits, unregular physical activity),^6^ short sleep duration and daytime napping^56^ with higher risk of the both IBD subtypes, and positive associations between intake of sugar-sweetened beverages, ultra-processed foods and higher risk of CD but not UC. Although genetic studies have reported overlapped genetic risk alleles, disease—specific alleles still exist for UC and CD, which might mediate disease-specific gene-environmental effects.^40^ Overall, the mechanisms underlying the disparity of the effect of air pollutants on UC and CD were unclear enough and merited further investigation.

Specific air pollutants such as PM, O_3_, NO_2_, and polycyclic aromatic hydrocarbons have been reported to influence DNA methylation of genes related to multiple chronic diseases.^25^ By utilizing genetic instrumental variables of DNA methylation markers associated with NO_2_ as well as PM_2.5_ and building upon the largest IBD GWAS, our MR analyses identified significant associations between modified methylation at multiple air pollution-related CpG sites and the susceptibility of UC. The roles of *CXCR2* and MHC class III region loci were further validated when we compared the findings with genome-wide methylation analysis of UC and conducted colocalization analysis. Importantly, we also examined the associations between DNA methylation and gene expression in colonic tissue and performed a single cell RNA sequencing analysis to investigate the expression of these two genes in distinct cell types of the intestinal tissues.

CXC motif chemokine receptor 2 (CXCR2) is the common receptor of CXCL1, CXCL5 and CXCL8, which are pro-inflammatory cytokines expressed in IBD. Consistently, a genome-wide DNA methylation study conducted using colon mucosal biopsies from treatment-naïve patients with UC and controls has confirmed our finding regarding *CXCR2*. The study revealed differential expression of *CXCR2* in the mucosa of patients with UC and its association with observed differential DNA methylation (*P* = 0.016).^57^ *CXCR2* has been demonstrated to be essential for the migration of neutrophils after air pollutant exposure.^58^ As for the role of *CXCR2* in UC, data based on samples from populations^59^ and animal models^60^ have reported increased expression of *CXCR2* in active colonic IBD and support the hypothesis of the important pathogenic role of *CXCR2+* neutrophils in the development of colitis.

Besides *CXCR2*, we discovered the roles of MHC class III region on associations between air pollution and incident UC. The MHC region has been extensively researched in the human genome due to its significant associations with autoimmune and inflammatory diseases, making it one of the most studied region in this field.^61^ Interestingly, early linkage and association studies had indicated that the region might have a more significant impact on susceptibility to UC than CD,^62^ with specific allelic associations being reported and validated for disease severity and extent. Goyette et al. conducted the most comprehensive analysis to date, which involved SNP typing of the MHC in over 32,000 individuals with IBD. Their findings demonstrated the presence of multiple human leukocyte antigen (HLA) alleles, with a predominant role observed for HLA-DRB1∗01:03 in both UC and CD.^63^ A recent study has also identified a distinct DNA methylation pattern in the MHC region of the sigmoid colon in patients with IBD.^64^ Further studies are needed to explore the interplay of genetic and epigenetic variation across the MHC III region, and to elucidate the mechanisms involved in the associations of these variations with the exposome in the pathogenesis of IBD and other autoimmune diseases. The close clustering of genes and linkage disequilibrium across the region are important considerations in study design and interpretation.

In addition to the *CXCR2* and MHC class III region loci, a series of genes including *SMAD3, P4HA2, NCR3, GPX1, EGFL8, CEBPA, DDAH2, LIME1* were discovered in the first stage of our study; it is possible that these loci might also be involved in linking air pollution and the risk of UC, although they were not validated in the additional analyses. Gene sites such as *SMAD3*^65^ have been identified by previous studies as IBD susceptibility loci, which might strengthen their potential role in the disease. Of interest, *GPX-1* is an intracellular antioxidant enzyme, limiting the harmful effects of hydrogen peroxide,^66^ thus emphasizing its role in modulating cellular oxidant stress. In the mouse, combined deficiency of *GPX1* and *GPX2* pre-disposes to ileocolitis.^67^^,^^68^ Notably, the NO_2_-related methylated CpG sites cg07462448 and cg24084564 have been validated in an epigenome-wide analysis of the association between air pollution and DNA methylation, which showed positive associations between methylation status at these determinants and NO_x_ as well as light-absorbing carbon in the multi-ethnic US born-participants.^69^

Importantly, smoking has been found to be associated with alterations of methylation at *CXCR2*, *SMAD3* and *GPX1*^70^, ^71^, ^72^ and loci at MHC class III region.^9^^,^^73^ *CXCR2* has been demonstrated to play an essential role in smoke-induced inflammation, and inhibition of *CXCR2* can reduce neutrophilic infiltration and tissue damage.^71^ In addition, cigarette smoke leads to the increase of *WNT-5B* expression, thus increasing *TGF-β/SMAD3*^72^ signalling in airway epithelial cells. Interestingly, epigenetic changes in *LTA/TNF* loci, located within the MHC III class region, were also found to play an important role in the association between smoking and CD risk.^9^ This suggests that the DNA methylation within MHC III class region may be influenced by multiple factors and involved in both pathogenesis of UC and CD. Pathways involving secretory vesicles, lysosome, leukocyte activation, and a series of immunity responses were identified in our gene ontology analysis. Overall, investigation of ontology of CpG sites related to NO_2_ exposure, along with analyses of transcriptomic correlates of MR-identified CpG sites, highlighted the broad impact of air pollution on immunity. Furthermore, an RCT study offers valuable insights for guiding the future direction of intervention research in patients with IBD, building upon the current findings.^32^

Our study has several strengths. This is a comprehensive study to explore the potential causal associations between air pollutants and the risk of IBD, and potential mechanisms from the perspective of DNA methylation. Its design triangulated the evidence across observational and genetic analyses, and across samples from colon tissue providing robust evidence for the mediating effects of DNA methylation on air pollution and UC. Of note, there are certain limitations in our current study. First, given the specific cohort under investigation, our study focused exclusively on participants of European ancestry and older age, thus limiting the generalizability of our findings to other populations. Further research is required to confirm these findings, necessitating the inclusion of diverse populations due to variations in methylation patterns among ethnic sub-groups.^74^ Secondly, although the LUR model has been recognized as an effective approach for estimating air pollution exposure, particularly for NO_2_ and NO_X_, there may still be potential misclassification. This could be attributed to the fact that the simplistic dispersion assumption of the LUR model is more suitable for traffic emissions rather than industrial point emissions. Additionally, factors such as the number of monitoring sites and the complexity and size of urban environments can also influence its predictive accuracy.^35^ Nevertheless, these challenges can be addressed through the implementation of an MR design which is less susceptible to environmental influences.

Although our MR analysis compensated for the bias of observational studies, pleiotropy may exist when using single nucleotide polymorphisms as instrumental variables. In the analyses for testing horizontal pleiotropy, we found no horizontal pleiotropy for PM_2.5_ but we detected horizontal pleiotropy for the analysis of NO_2_. It is important to note that we studied one instrument variable for most of air pollution related CpG sites, and we have not been able to assess these results by performing multivariable MR and other sensitivity analyses. However, we have supplemented our data with a series of validation analyses to confirm the findings.

In addition, we acknowledge that the evaluation of air pollution and the associated changes in DNA methylation CpG sites relied on cross-sectional data, thereby lacking longitudinal monitoring of exposure and the dynamic changes of exposure on methylation. Finally, although there is evidence suggesting that certain methylation markers in the blood may reflect corresponding methylation signatures in intestinal tissues,^75^ it should be noted that DNA methylation characteristics vary among different cell types and tissues.^76^ That means that the current study has not directly investigated the altered DNA methylation patterns in intestinal tissues induced by air pollution. Again, we have substantiated the findings related to these two target genes through a series of additional analyses. These includes an investigation into the regulatory pattern of DNA methylation on gene expression in colonic tissues, querying the GTEx Portal, and conducting single cell RNA sequencing analysis on distinct intestinal cells. Furthermore, the key findings have biological plausibility at several levels; the discovery regarding *CXCR2* has also been corroborated by a previous genome-wide DNA methylation study conducted on mucosal samples^57^ Biomarkers identified in both blood and intestinal tissue serve as complementary tools for identifying diagnostic and treatment targets for the disease. The above issue should be more directly addressed in future studies, particularly with the availability of specific EWAS of air pollution conducted across various tissues and cell types.

In summary, our study provides an exciting research paradigm for future studies that aim to explore potential mechanisms of environment-IBD by integrating observational, genetic and methylation evidence and highlights the importance of epigenetic alterations in complex disease pathogenesis of UC. We report a potential causal association between air pollution and risk of UC, but not CD. We also provide evidence for biological mechanisms for this association, specifically the epigenetic alteration of *CXCR2* and determinants within the MHC class III region. The findings of epigenetic dysregulation linking air pollution to the risk of UC demonstrate the translational potential of these techniques in defining biomarkers as well as characterizing pathways for pharmacological interventions.

## Contributors

Jie Chen, methodology (lead), formal analysis (equal), writing-original draft (equal); Han Zhang, methodology (equal), formal analysis (lead), writing-original draft (equal); Tian Fu, methodology (equal), formal analysis (equal), writing-original draft (lead); Jianhui Zhao, methodology (supporting), writing-original draft (supporting), writing-review and editing (supporting); Jan Krzysztof Nowak, formal analysis (equal), conceptualization (equal), supervision (equal); Rahul Kalla, methodology (equal), writing-review and editing (equal); Judith Wellens, methodology (equal), writing-review and editing (lead); Shuai Yuan, methodology (supporting), writing-review and editing (supporting); Alexandra Noble, methodology (equal), writing-review and editing (supporting); Nicholas T Ventham, conceptualization (equal), writing-review and editing (supporting); Malcolm Dunlop, writing-review and editing (supporting), methodology (equal); Jonas Halfvarson, conceptualization (supporting), writing-review and editing (supporting); Ren Mao, methodology (equal), writing-review and editing (equal), supervision (equal); Evropi Theodoratou, conceptualization (lead), supervision (supporting), writing-review and editing (equal); Jack Satsangi, conceptualization (lead), supervision (equal), writing-review and editing (equal); Xue Li, conceptualization (lead), methodology (lead), supervision (lead), writing-review and editing (supporting). Jie Chen, Han Zhang, Tian Fu, and Xue Li have directly accessed and verified the underlying data reported in the manuscript. Evropi Theodoratou, Jack Satsangi, and Xue Li are the study guarantors. All authors read and gave final approval of the version to be published.

## Data sharing statement

The results of this study are included in this article and the Supporting files. The UK Biobank is an open access resource and researchers required approval from the UK Biobank (www.ukbiobank.ac.uk/). Genetic data was derived from the large genome-wide association study (GWAS), Genetics of DNA Methylation Consortium (GoDMC) and genome-wide DNA methylation analyses, which can be required from the published articles.

## Declaration of interests

JKN reports consulting for Procter & Gamble and grant support from the Biocodex Microbiota Foundation, outside of the submitted work. All the remaining authors declare no potential or actual conflict of interest to the work presented in this paper.
