# Supplementary material for: Exposure to air pollution increases susceptibility to ulcerative colitis through epigenetic alterations in CXCR2 and MHC class III region
Source: eBioMedicine. 2024 Nov 13;110:105443. doi: 10.1016/j.ebiom.2024.105443 (PMC11605448; doi:10.1016/j.ebiom.2024.105443)
Supplement: Supplementary Figures [file mmc1.pdf]

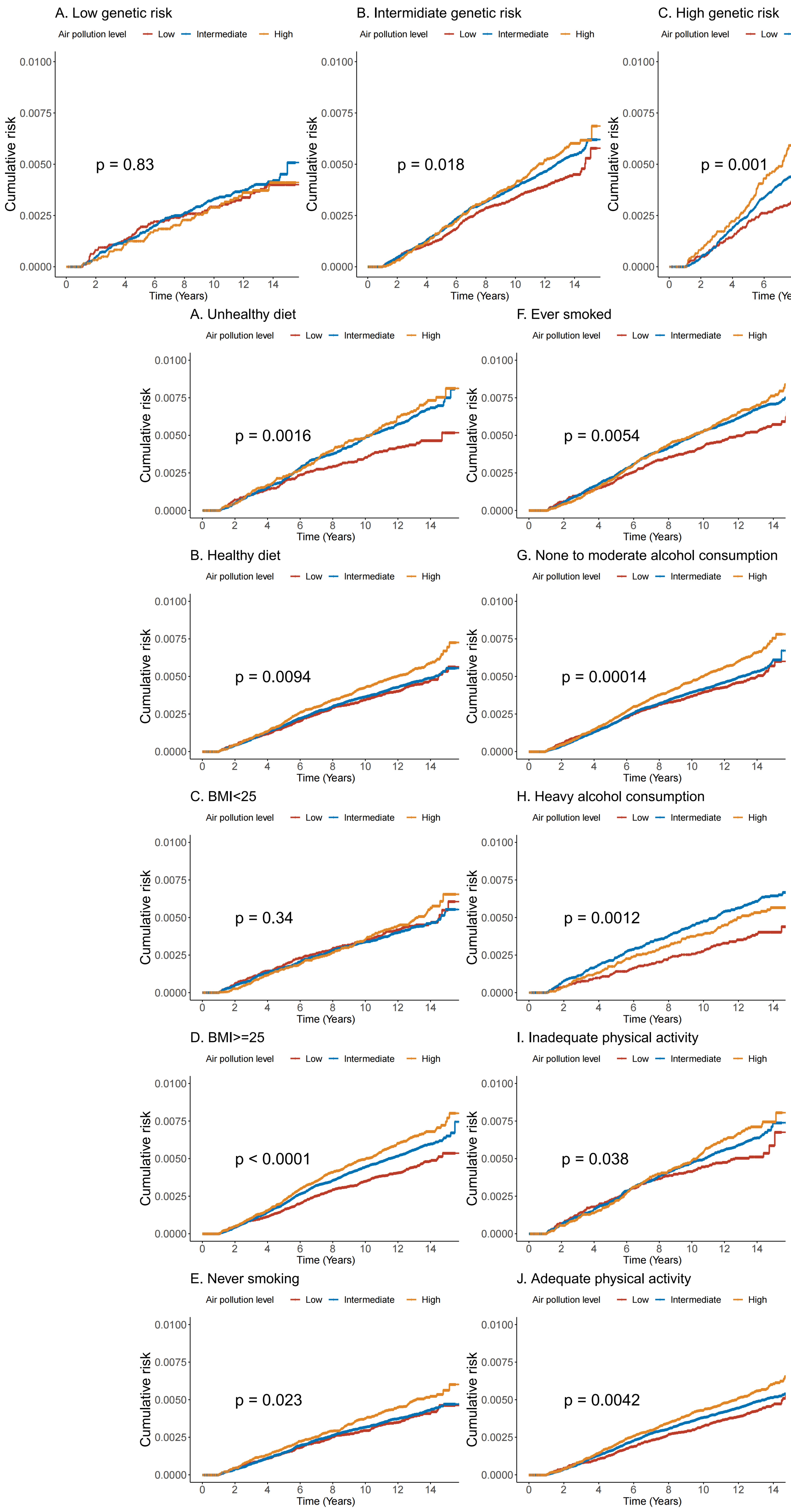

Figure S1. The Kaplan-Meier plots for the associations of different levels of air pollution with the risk of UC in subgroups according to genetic risk and lifestyle.

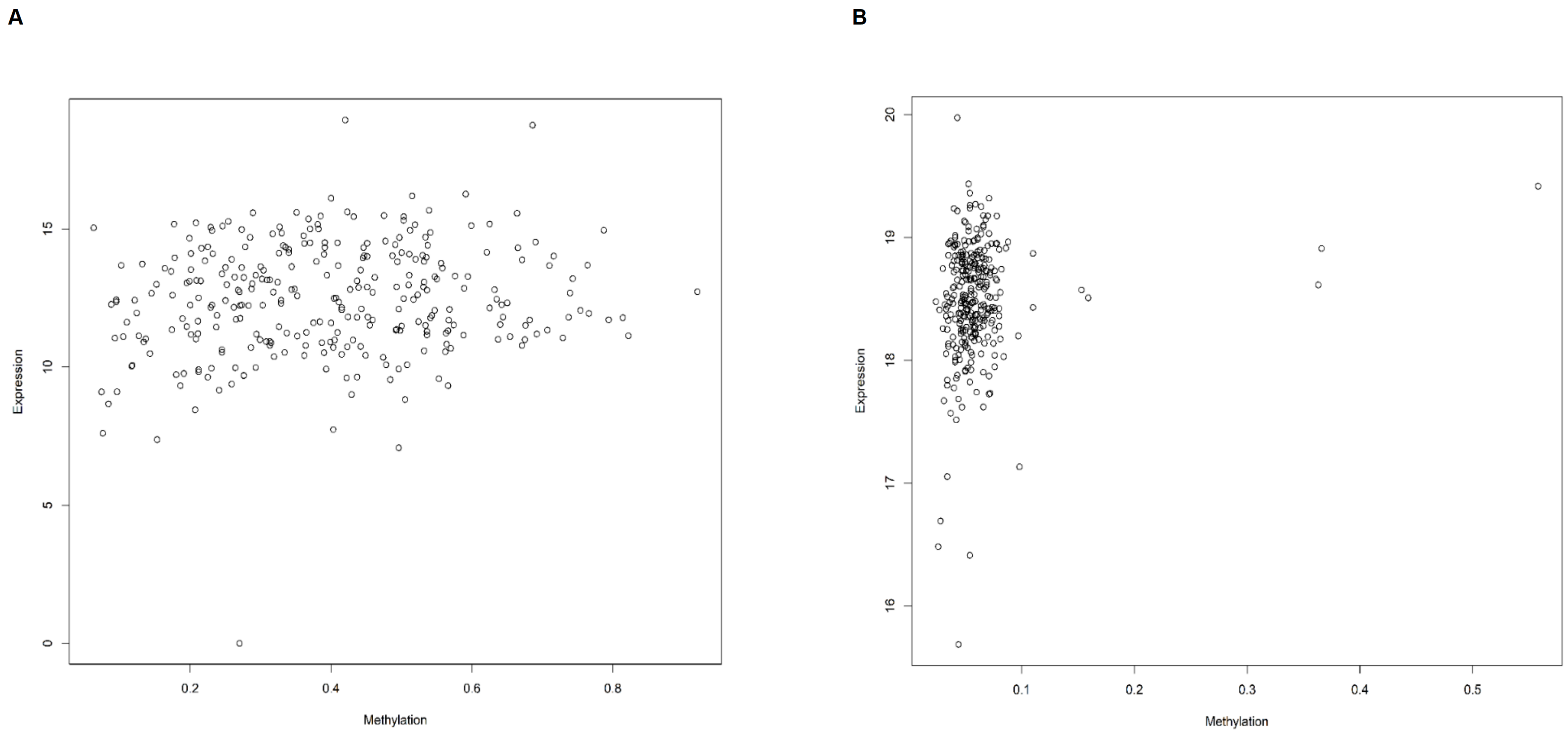

Figure S2. The scatter plot for the DNA methylation and gene expression profiles in colon tissues.
A, DNA methylation at cg06547715 and the expression of CXCR2.
B, DNA methylation at cg16689962 and the expression of AGPAT1.

(A)

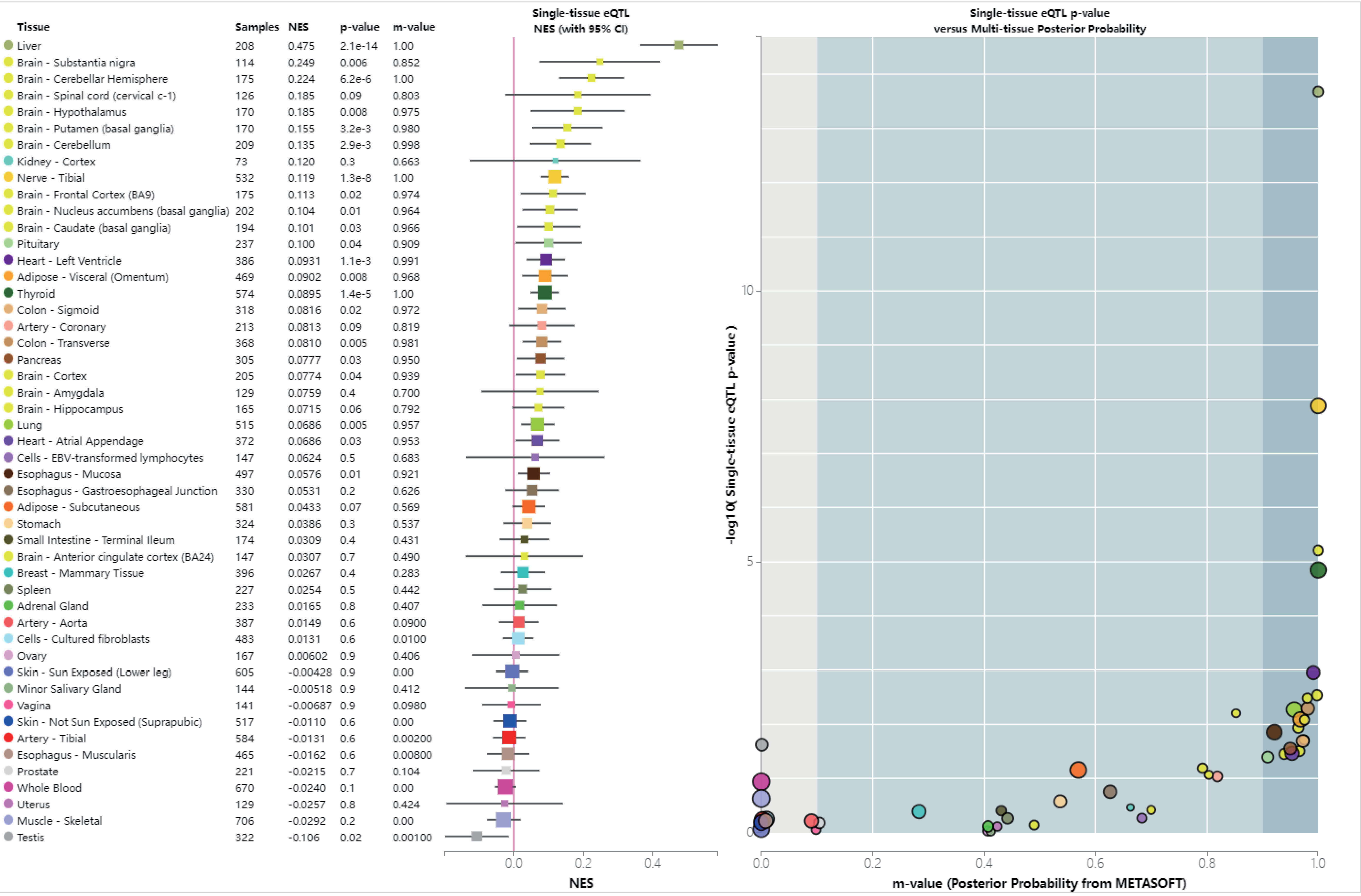

(B)

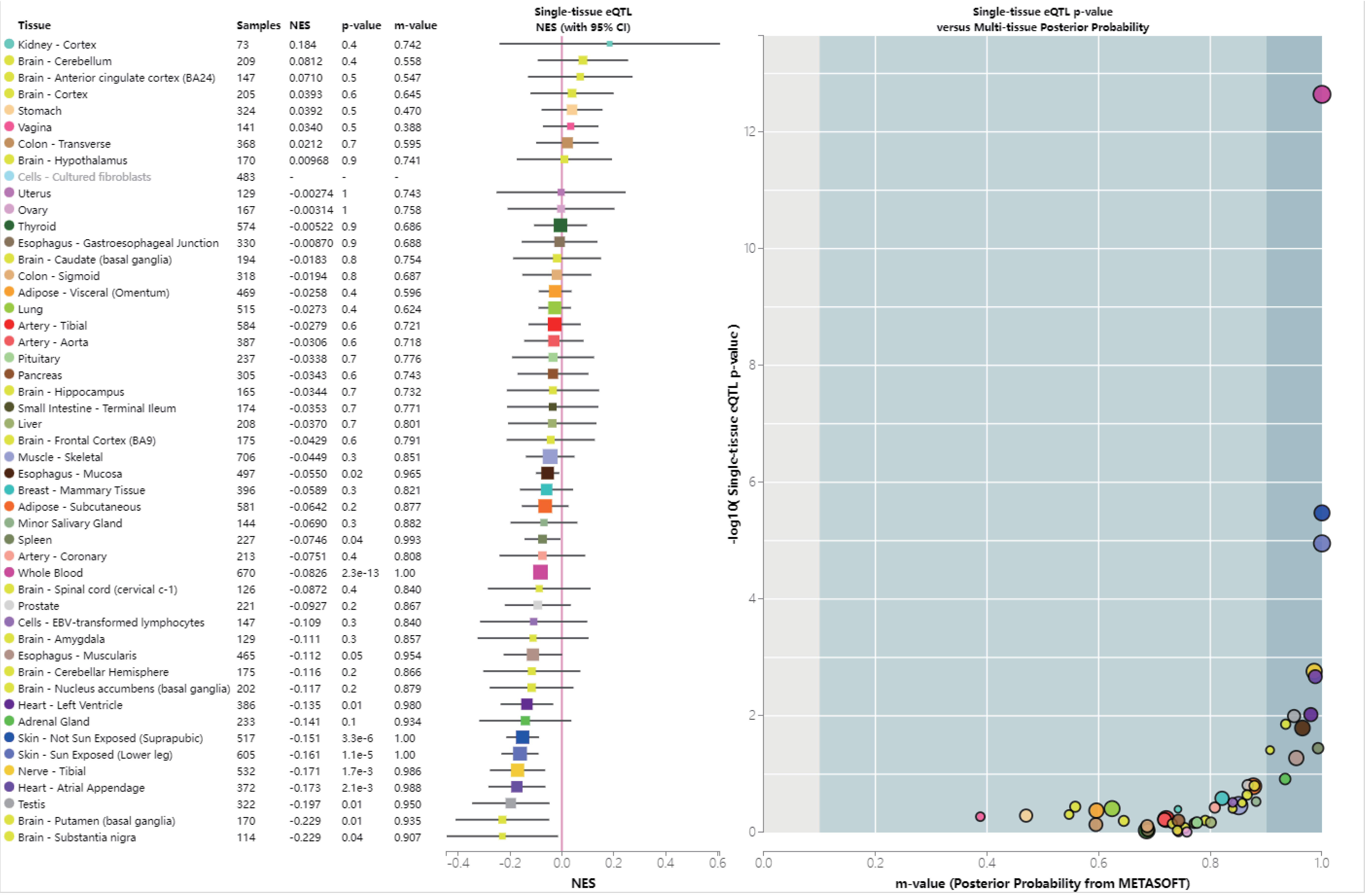

Figure S3. Single-tissue eQTL plot of rs3132934 (A, cg16689962, AGAPT1) and rs4133195 (B, cg06547715, CXCR2).

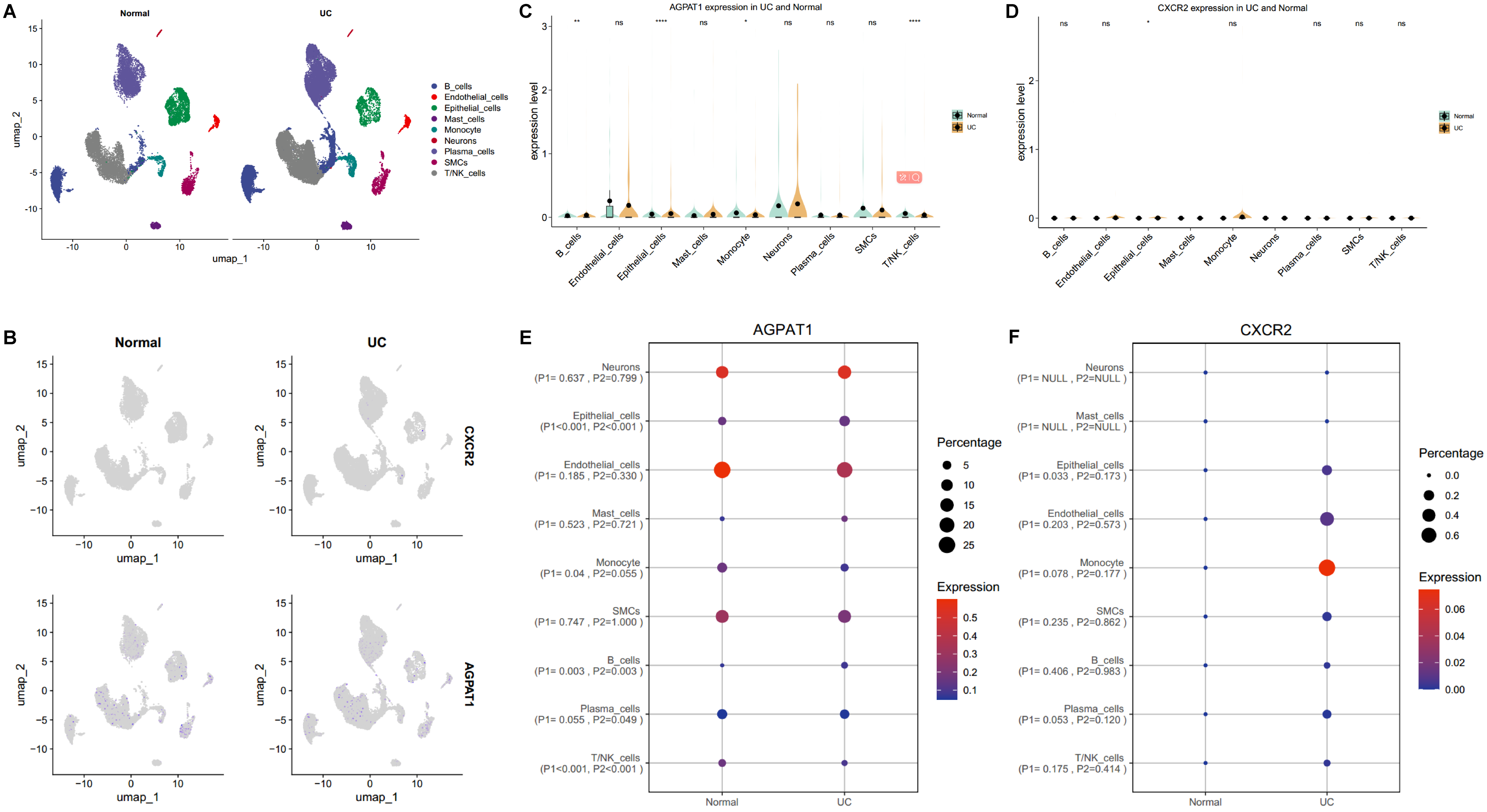

Supplementary Figure 4. The single cell RNA sequencing analysis of AGPAT1 and CXCR2 in intestinal cells. A, the annotation of cell types in intestinal. B, the global expression of AGPAT1 and CXCR2 in intestinal. C, the expression of AGAPT1 in specific intestinal cells. D, the expression of CXCR2 in specific intestinal cells. E, the percentage and expression differences of AGPAT1 in specific cells. F, the percentage and expression differences of CXCR2 in specific cells.
